# From work-unit member to social individual: Retirement transition patterns, leisure reconfiguration, and depressive symptom trajectories in China

**DOI:** 10.1097/MD.0000000000050256

**Published:** 2026-08-14

**Authors:** Lanka Wu

**Affiliations:** a School of Tourism and Hotel Management, University of Sanya, Sanya, Hainan, China.

**Keywords:** aging, china, depression trajectories, leisure activity, life course, retirement transition, work-unit system

## Abstract

The dissolution of China’s work-unit system has reshaped retirement, but how transition patterns relate to long-term depressive symptom trajectories and whether leisure reconfiguration mediates this relationship remain unclear. To examine the longitudinal association between retirement transition patterns and depressive symptom trajectories in later life and the mediating role of leisure reconfiguration, data were collected from the Northeast China Aging and Lifestyle Survey (NCALS, N = 5217, age ≥ 60 years). Retirement transition patterns (gradual, abrupt, nonstandard) and leisure changes were assessed retrospectively at baseline. Depressive symptoms were measured prospectively in 3 waves (GDS-15). Growth mixture modeling determined symptom trajectories. Multivariable logistic regression and structural equation modeling examined associations and mediation and adjusted for covariates. Four depressive symptom trajectories occurred: stable low (48.3%), gradual increase (32.7%), high fluctuation (12.5%), and late-onset surge (6.5%). Compared with gradual transition, abrupt stop (adjusted odds ratio [aOR] = 2.87, 95% CI: 2.24–3.68) and nonstandard transition (aOR = 3.45, 2.68–4.44) were associated with higher odds of the late-onset surge trajectory. Leisure reconfiguration mediated 31.7% of the associations (17.1% for travel diversity). The association between low social support (*P* interaction = .008) and low pension was stronger. In this observational study, gradual retirement through leisure reconfiguration was associated with a healthier trajectory of depressive symptoms. The findings highlight the importance of recalled transitional experiences for mental health and support phased retirement and leisure participation policies, although causal inference is limited by retrospective assessment.

## 1. Introduction

Population aging poses profound challenges to societies worldwide, necessitating a change in thinking from viewing later life merely as a period of decline to understanding the factors that promote resilience and well-being.^[1]^ In China, this demographic transition is uniquely intertwined with a profound institutional change, namely, the gradual disintegration of the work-unit system. This system once organized not only employment for urban residents but also their housing, healthcare, and social life.^[2]^ For generations, the work unit was the cornerstone of identity, providing a lifelong sense of social role and collective purpose. Retirement from the system, therefore, is more than just a withdrawal from the labor market. It marks a fundamental break with the deeply embedded “work-unit member” identity and a challenging transition to an autonomous “social individual” identity.^[3]^ This historical context provides a critical lens through which to examine the diversity of retirement experiences and their long-term impact on mental health.

Retirement is a major turning point in the life course that can trigger dramatic changes in daily routines, social networks, and self-perceptions. While earlier theories portrayed retirement as a uniform crisis, contemporary research acknowledges it as a heterogeneous process with varying implications for psychological well-being.^[4]^ The concept of retirement transition patterns, which capture differences in the timing, voluntariness, and gradualness of retirement, has received increasing attention as a key predictor of post-retirement adjustment.^[5]^ A gradual or phased transition may allow individuals to engage in anticipatory socialization, experiment with new roles, and gradually develop new routines and identities, thus alleviating the pressure caused by the sudden loss of roles.^[6]^ In contrast, an involuntary or abrupt cessation of work may lead to a sudden loss of daily structure and social contacts, increasing vulnerability to depressive symptoms.^[7]^

Despite these conceptual advances, critical research gaps remain. First, the role of post-retirement leisure activities, particularly their reconfiguration rather than mere volume, as a potential mechanism linking transition patterns to mental health is understudied.^[8]^ Leisure is not a passive outcome but an active domain for identity reconstruction and social re-engagement.^[9]^ According to continuity theory, individuals strive to maintain their previous patterns of activity and social ties to preserve a sense of self.^[10]^ Therefore, successful reconfiguration of leisure pursuits, such as shifting from work-centered socializing to travel-based exploration or community volunteering, may be critical to well-being in retirement.^[11]^ Second, most studies on retirement and mental health rely on cross-sectional designs or short-term follow-up, which fail to capture the trajectory of dynamic depression that may last for years.^[12]^ Some people may show resilience, others may show gradual decline, and others may show fluctuating or delayed patterns of distress.^[13]^ Identifying these different developmental paths and their antecedents is essential for targeted prevention. Third, the unique socio-historical context of the post-work-unit era in China remains largely unintegrated into mainstream gerontological theory.^[14]^ The type of work unit, such as state-owned enterprises and the private sector, may not only affect pension security but also shape people’s understanding of the meaning of retirement and the resources available to cope with retirement.^[15]^

This study aims to fill these gaps by integrating life course epidemiology, social gerontology, and sociological institutionalism.^[16]^ Using longitudinal data from Northeast China, the hinterland of the old industrial work-unit system, this observational study investigated: longitudinal associations between data-based, retrospectively reported retirement transition patterns and subsequent late-life depression symptoms; the extent to which the retrospectively reported reconfiguration of leisure activities in retirement mediates this association; and whether social support and economic resources moderate these relationships. It was hypothesized that a retrospective report of a gradual retirement transition would be associated with membership in more resilient symptom trajectories and that this association would be partially mediated by a broader and more diverse reconfiguration of self-reported leisure activities. By highlighting the institutional heritage of the work-unit system, this study explores the relationship between macro-social transformation and the mental health trajectory of the aging population from a contextualized and focused perspective.

## 2. Methods

### 2.1. Study design and data source

This observational study used data from the Northeast China Aging and Lifestyle Survey (NCALS). Baseline data were used to retrospectively assess retirement transition patterns and post-retirement leisure activity reconfiguration and to examine their associations with depressive symptom trajectories identified through 3 rounds of prospective follow-up. NCALS is a population-based prospective study initiated in 2018 to investigate the social, behavioral, and environmental determinants of healthy aging in Liaoning, Jilin, and Heilongjiang provinces. A representative sample of community-dwelling older adults aged 60 years and older was recruited using multistage stratified random cluster sampling. The baseline survey (first round, 2018–2019) included comprehensive face-to-face interviews, physical measurements, and cognitive assessments. Follow-up surveys are conducted approximately every 2 years (second round, 2020–2021; third round, 2022–2023). The Institutional Review Board of Sanya University approved the study, and all participants provided written informed consent.

### 2.2. Study population

**Figure 1.**
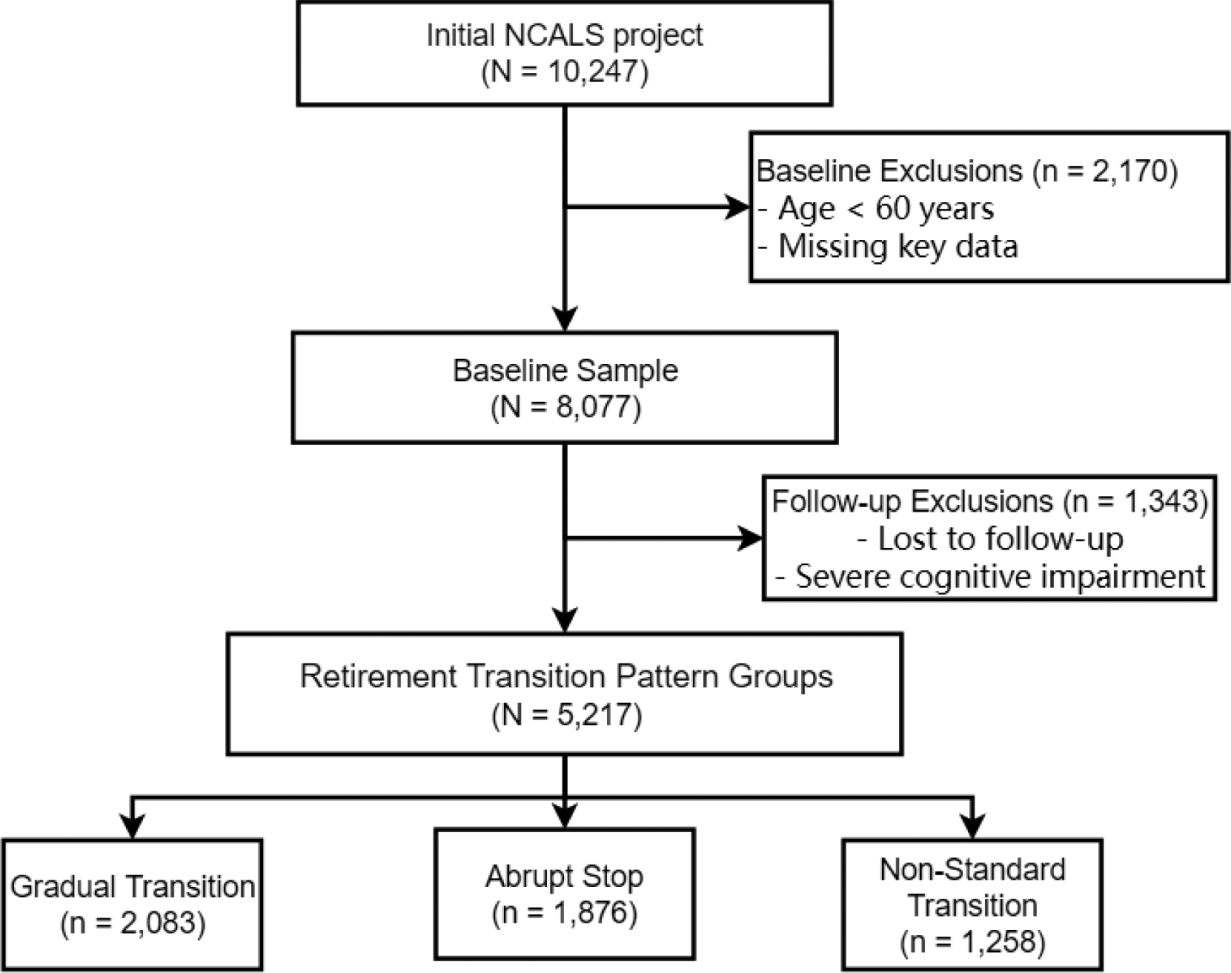
Study participant flow diagram of the Northeast China Aging and Lifestyle Survey (NCALS). The flowchart illustrates the inclusion and exclusion process of participants in the current analysis. From the initial NCALS database (N = 10,247), participants were excluded at baseline (n = 2170) due to age < 60 yr or missing key data. The baseline sample (N = 8077) underwent a 2-yr follow-up, with additional exclusions (n = 1343) for loss to follow-up or severe cognitive impairment. The final analytic sample (N = 5217) was categorized into 3 retirement transition pattern groups based on their retrospectively reported retirement characteristics at baseline: Gradual transition (n = 2083), abrupt stop (n = 1876), and non-standard transition (n = 1258). The analytic sample differed from those excluded on several baseline characteristics (e.g., younger age, lower baseline depression), indicating potential selection bias.

From the initial NCALS cohort (first round N = 10,247), participants who met the following criteria were included in this analysis: had formally retired from their primary lifetime employment; had complete retrospective data on the characteristics of retirement transitions, including timing, voluntariness, and progressivity; they participated in at least 2 rounds of depression assessment; and there was no diagnosis of dementia or severe cognitive impairment (mini-mental state examination score < 18) at baseline. Participants who were never formally employed or whose key data were missing by more than 10% were excluded. The final analysis sample comprised 5217 individuals. The baseline characteristics of the analytic sample were compared with those of participants excluded due to loss to follow-up or missing data (n = 2860). The analysis sample was slightly younger, had higher education, better socioeconomic status, and lower baseline depressive symptoms (all *P* < .05), suggesting potential selection bias. A detailed participant flow chart is shown in Figure 1.

### 2.3. Measures

#### 2.3.1. Primary exposure: retirement transition pattern

The exposure variable was constructed using the life course calendar method administered at baseline, which collected detailed retrospective information on employment history.^[17]^ This method relies on participant recall and may be biased. According to the existing literature,^[18]^ the retirement transition is characterized by 3 dimensions.

**Gradualness:** Coded as Abrupt (full-time work directly to full retirement) or Gradual (reduced work hours, part-time work, or consulting for ≥6 months before full retirement).
**Voluntariness:** Based on self-report, coded as voluntary (“chose to retire when I did”) or involuntary (“had to retire due to health, layoffs, or mandatory policy”).
**Timing:** Age at retirement, categorized relative to the official pension age (Male: 60; Female: 50/55) as early (>5 years before), on time (±5 years), or late (>5 years after).

These 3 dimensions were synthesized using latent class analysis to derive empirically defined transition patterns.^[19]^ The optimal three-class solution identified:

**Class 1:** Gradual transition (GT): Characterized by gradual withdrawal, voluntary decision, and on time/late retirement.
**Class 2:** Abrupt stop (AS): Characterized by abrupt cessation, mixed voluntariness, and on-time retirement.
**Class 3:** Non-standard transition (NST): Characterized by abrupt cessation, involuntary decision, and early retirement.

#### 2.3.2. Mediator: post-retirement leisure activity reconfiguration

Leisure activity was assessed at wave 1 (post-retirement for all) via a validated questionnaire. Reconfiguration was operationalized as the self-reported degree of positive change in leisure participation after retirement compared to the 5-year pre-retirement period (assessed retrospectively). It was a composite latent construct derived from 3 domains, each scored from 0 (decreased) to 2 (significantly increased)^[20]^:

**Travel diversity:** Number of diverse types of leisure trips taken (e.g., cultural, nature, family visits) and frequency.
**Social activity participation:** Frequency of participation in group-based activities (e.g., community clubs, group exercise, volunteering).
**New skill acquisition:** Engagement in learning new hobbies or skills (e.g., painting, musical instruments, technology use).

Confirmatory factor analysis supported a single-factor structure (comparative fit index CFI = 0.95, root mean square error of approximation RMSEA = 0.04), and a composite score was used in the analysis. This retrospective assessment of change is susceptible to recall bias and current state (e.g., current mood affects perception of past change).

#### 2.3.3. Outcome: trajectories of late-life depression

Depressive symptoms were measured in the first, second, and third rounds using the 15-item geriatric depression scale (GDS-15), a validated instrument with scores ranging from 0 to 15.^[21]^ The outcome was operationalized as trajectories of depressive symptoms, recognizing that the GDS-15 is a screening instrument rather than a diagnostic tool for clinical depression. To model the longitudinal course of depression, growth mixture modeling was employed in Mplus 8.3.^[22]^ The growth mixture model can identify unobserved subpopulations that follow different trajectories over time. Models with 1 to 5 classes were estimated. The optimal number of classes is determined by evaluating the Bayesian Information Criterion, the sample size corrected Bayesian Information Criterion, the entropy value (≥0.8 indicates a good classification), the Lo–Mendell–Rubin adjusted likelihood ratio test, and the theoretical interpretability.

#### 2.3.4. Covariates

Based on a directed acyclic graph constructed from prior literature,^[23]^ the following baseline (wave 1) variables were included as potential confounders:

**Sociodemographics:** Chronological age, sex, education level (primary, secondary, college+), current urban/rural residence, marital status.
**Socioeconomic & work history:** Pre-retirement work unit type (State-owned enterprise, Government/Institution, Collective enterprise, Private/Other), household asset index (quintiles), personal pension income (RMB/month).
**Health status:** Number of physician-diagnosed chronic conditions (from a list of 12), body mass index, baseline cognitive function (mini-mental state examination score).
**Psychosocial factors:** Perceived social support score (12-item scale), loneliness score (3-item University of California, Los Angeles Loneliness Scale scale).

### 2.4. Statistical analysis

Descriptive statistics were calculated for the overall sample and for samples stratified by retirement transition category, and differences between groups were assessed using analysis of variance for continuous variables and chi-square tests for categorical variables. The analysis process involved first growth mixed modeling to identify different depressive symptom trajectories through repeated measures of GDS-15 scores, followed by an examination of their association with retirement transition patterns using multivariable logistic regression, adjusted for covariates. To explore the underlying mechanism, structural equation modeling was used to test whether the reconfiguration statistically mediated this association. Mediation was assessed by estimating the indirect effect (a × b) with bootstrapped 95% confidence intervals (5000 resamples), adjusted for age, sex, education, household assets, social support, and baseline health status.^[24]^ It is important to point out that this mediation analysis is based on a cross-sectional assessment of exposure and mediation relationships and is therefore observational in nature, limiting causal inferences about mediation pathways. Finally, the potential effects of social support and pension level were examined by introducing interaction terms in the regression model. Collinearity among covariates was assessed using variance inflation factors, with all values below 2.5, indicating no substantial multicollinearity.

Additional sensitivity analyses were conducted: first, the inverse probability weighting method was used to balance the baseline characteristics across the retirement transition groups.^[25]^ Second, *E* values were calculated to assess the potential impact of unmeasured confounders on the association of primary exposures with outcomes.^[26]^ Missing data for covariates (<5% for any single variable) were handled using multiple imputations by chained equations with 20 imputed datasets. All analyses were performed using R version 4.3.0, with a two-sided *P* value of <.05 indicating statistical significance.

## 3. Results

### 3.1. Study population and baseline characteristics

The final analysis sample included 5217 older adults with complete data on retirement transition patterns and follow-up depression symptoms. Based on their retrospectively reported retirement transition patterns, participants were divided into 3 groups: GT (n = 2083), an AS group (AS, n = 1876), and a nonstandard retirement group (NST, n = 1258). Baseline characteristics stratified by retirement transition pattern are detailed in Table 1. All sociodemographic, socioeconomic, health, and psychosocial variables showed significant gradient differences across groups (*P* < .001 except for sex *P* = .042). Participants in the GT group were the youngest, had the highest education and socioeconomic status, the best self-rated health, the highest level of physical activity, and the strongest social support. In contrast, the NST group exhibited the most unfavorable characteristics in each of these areas. These systematic differences suggest that retirement transition patterns are highly endogenous and are associated with widespread preexisting strengths or weaknesses. Multivariable adjustment and sensitivity analyses (inverse probability weighting) were employed to address this confounding, but residual confounding may still be present.

**Table 1:** Baseline characteristics of the study population by retirement transition pattern (N = 5217).

Characteristic	Overall (n = 5217)	Gradual transition (n = 2083)	Abrupt stop (n = 1876)	Non-standard transition (n = 1258)	*P* value
Sociodemographics					
Age, yr, mean ± SD	71.3 ± 6.8	69.9 ± 5.9	72.4 ± 6.8	73.0 ± 7.2	<.001
Female, %	56.8	54.2	58.1	58.9	.042
Education, %					
Primary or below	42.7	35.4	46.3	50.2	<.001
Secondary	39.2	42.1	38.7	36.4	
College or above	18.1	22.5	15.0	13.4	
Pre-retirement work unit type, %					
State-owned enterprise	51.3	58.2	48.7	42.1	<.001
Government/Institution	18.4	22.5	16.3	14.8	
Collective enterprise	14.2	9.8	17.4	18.9	
Private/Other	16.1	9.5	17.6	24.2	
Socioeconomic status					
Household asset index (1–5), mean ± SD	3.12 ± 1.37	3.58 ± 1.24	2.87 ± 1.31	2.56 ± 1.42	<.001
Pension ≥ 3000 RMB/mo, %	38.4	51.2	29.8	24.3	<.001
Health status & behaviors					
Number of chronic conditions, mean ± SD	2.34 ± 1.58	2.01 ± 1.42	2.57 ± 1.63	2.72 ± 1.71	<.001
Moderate-to-vigorous pa, min/wk, mean ± SD	108.7 ± 94.3	132.5 ± 102.4	92.8 ± 86.7	85.4 ± 82.1	<.001
Current smoker, %	22.7	19.4	24.8	26.3	<.001
Psychosocial factors					
Social support score (3–12), mean ± SD	8.82 ± 2.41	9.54 ± 2.12	8.37 ± 2.38	7.91 ± 2.57	<.001
Loneliness score (3–9), mean ± SD	4.71 ± 1.82	4.28 ± 1.64	4.92 ± 1.85	5.24 ± 1.93	<.001
Baseline depressive symptoms (GDS-15 ≥ 5), %	28.4	22.7	31.9	35.2	<.001

Note: The retirement transition pattern was retrospectively categorized at baseline. Systematic differences across groups in nearly all baseline characteristics indicate that the transition pattern is strongly endogenous and associated with preexisting advantages (gradual transition group) or disadvantages (non-standard transition group). *P* < .001 for all comparisons except sex (*P* = .042).

SD = standard deviation, GDS-15 = 15-item geriatric depression scale.

### 3.2. Identification of late-life depression trajectories

**Figure 2.**
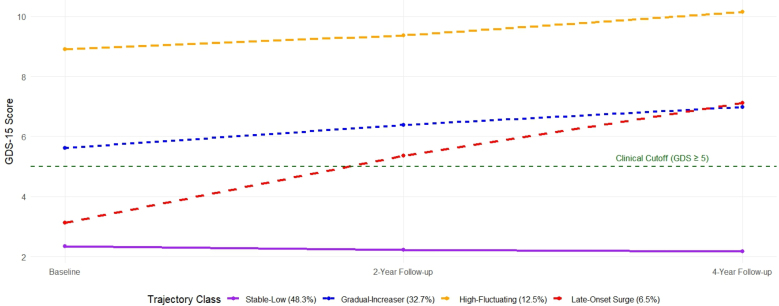
Latent class trajectories of late-life depressive symptoms identified by growth mixture modeling. Based on geriatric depression scale (GDS‑15) scores from 3 assessment waves (baseline, 2‑yr and 4‑yr follow‑ups), 4 distinct trajectories of depressive symptoms were identified: Stable‑low (48.3%), characterized by consistently minimal symptoms; gradual‑increaser (32.7%), with moderate baseline levels and a steady increase; high‑fluctuating (12.5%), showing high and variable symptom levels; and late‑onset surge (6.5%), with initially low scores followed by a sharp rise at the final follow‑up. The dashed horizontal line marks the clinical screening threshold (GDS‑15 ≥ 5). GDS-15 = 15-item geriatric depression scale.

Growth mixed model analysis was conducted based on the GDS-15 scores at 3 time points, and the four-category model was identified as the optimal model according to the fit index and interpretability (Table 2). Four trajectories were named: stable low-depressive type (48.3%), characterized by the maintenance of very low levels of depressive symptoms at all times; gradually rising type (32.7%), with moderate baseline levels and steady increases over time; high-fluctuating type (12.5%), with high levels of symptoms and marked fluctuations; and late-life sudden onset (6.5%), with a low initial score but a sharp rise in the latter part of the follow-up. The model shows good classification accuracy (entropy value = 0.83). The 4 depression trajectories identified are shown in Figure 2.

**Table 2:** Characteristics of latent class trajectories of late-life depressive symptoms identified by growth mixture modeling (N = 5217).

Parameter	Stable-low	Gradual-increaser	High-fluctuating	Late-onset surge
Prevalence, % (n)	48.3 (2521)	32.7 (1706)	12.5 (652)	6.5 (338)
Pattern description	Consistently low scores over time	Moderate initial level, steady increase	High baseline with significant fluctuation	Low initial level, sharp rise in late follow-up
Growth parameters (SE)				
Intercept	2.34 (0.21)	5.62 (0.32)	8.91 (0.48)	3.12 (0.28)
Linear slope	−0.08 (0.04)	0.42 (0.07)	0.15 (0.12)	1.24 (0.18)
Quadratic term	0.01 (0.002)	−0.02 (0.005)	0.04 (0.008)	−0.06 (0.011)
Model fit				
BIC	24,531.7	24,892.4	25,123.8	25,247.3
Entropy	0.87	0.83	0.79	0.81

Model was fitted based on GDS-15 scores from 3 repeated assessments (baseline, 2-yr, and 4-yr follow-ups). A lower BIC indicates better model fit. Entropy > 0.7 suggests acceptable classification accuracy.

BIC = bayesian information criterion, GDS-15 = 15-item geriatric depression scale.

### 3.3. Association between retirement transition patterns and depression trajectories

Multivariable logistic regression analysis showed a strong association between transition to retirement patterns and adverse depression trajectory attributions, using the stable low depression type as a reference (Table 3). After adjustment for sociodemographic variables, type of work unit, and health covariates, both the AS and NST groups had significantly higher odds of belonging to all adverse symptom trajectories compared with the GT group. The strongest associations were found for later-life burst-like trajectories (AS group: adjusted odds ratio (aOR) = 2.87, 95% confidence interval [CI]: 2.24–3.68; NST group: aOR = 3.45, 95% CI: 2.68–4.44). Pre-retirement employment in collective or private enterprises was also independently associated with higher odds of adverse trajectories compared with state-owned enterprises. Sensitivity analysis using inverse probability weighting yielded similar but slightly attenuated estimates (aOR = 3.12, 95% CI: 2.41–4.04 for late-life suddenness in the NST group compared with the GT group). The *E*-value for the observed association between the NST group and later-life suddenness trajectories was 5.64, with an *E*-value of 4.05 corresponding to the lower bound of the confidence interval. This suggests that an unmeasured confounder would need to be associated with both the exposure and the outcome by a risk ratio of at least 5.64 each, above and beyond the measured covariates, to fully explain away the observed association; a confounder associated by a risk ratio of 4.05 each could shift the confidence interval to include the null. Thus, weaker unmeasured confounding would be unlikely to eliminate the observed association, although it could attenuate the estimated effect.

**Table 3:** Multivariable logistic regression of retrospectively reported retirement transition patterns associated with depressive symptoms trajectory membership (reference: stable-low trajectory).

Variable	Gradual-increaser trajectory		High-fluctuating trajectory		Late-onset surge trajectory	
	aOR (95% CI)	*P*	aOR (95% CI)	*P*	aOR (95% CI)	*P*
Retirement transition pattern (ref: gradual transition)						
Abrupt stop	1.82 (1.54–2.15)	<.001	2.43 (1.98–2.98)	<.001	2.87 (2.24–3.68)	<.001
Non-standard transition	2.14 (1.78–2.58)	<.001	3.12 (2.52–3.86)	<.001	3.45 (2.68–4.44)	<.001
Work unit type (ref: state-owned enterprise)						
Government/Institution	0.87 (0.72–1.05)	.143	0.92 (0.73–1.16)	.491	0.85 (0.64–1.13)	.264
Collective enterprise	1.24 (1.03–1.49)	.024	1.38 (1.10–1.73)	.005	1.42 (1.08–1.87)	.012
Private/Other	1.53 (1.26–1.85)	<.001	1.79 (1.42–2.26)	<.001	1.94 (1.48–2.54)	<.001
Covariates						
Age (per 5-yr increase)	1.18 (1.10–1.27)	<.001	1.31 (1.20–1.43)	<.001	1.42 (1.28–1.58)	<.001
Female (vs Male)	1.15 (1.01–1.31)	.038	1.32 (1.13–1.54)	<.001	1.21 (1.00–1.46)	.051
Household asset index (per unit)	0.85 (0.80–0.90)	<.001	0.79 (0.73–0.85)	<.001	0.76 (0.69–0.83)	<.001
Social support score (per unit)	0.89 (0.86–0.92)	<.001	0.84 (0.80–0.88)	<.001	0.87 (0.82–0.92)	<.001

Note: Models were adjusted for all variables listed, as well as education, marital status, pension level, number of chronic conditions, physical activity, smoking, baseline loneliness, and baseline cognitive score.

aOR = adjusted odds ratio, CI = confidence interval.

### 3.4. Mediating role of leisure activity reconfiguration

**Figure 3.**
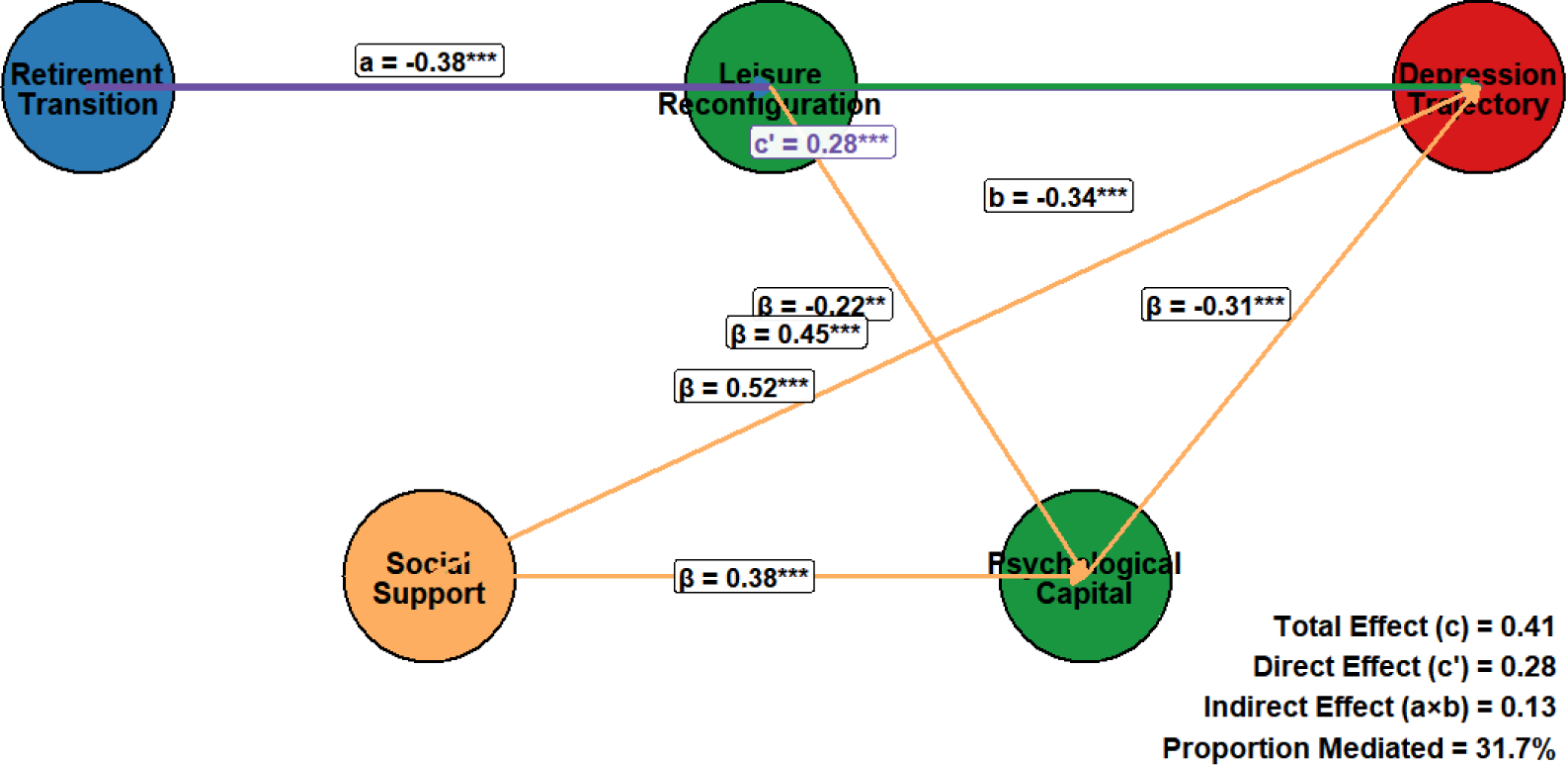
The path diagram illustrates the mediation effect of leisure activity reconfiguration. The model tests whether retrospectively self-reported post-retirement leisure activity reconfiguration is associated with the observed link between retirement transition patterns and adverse depressive symptom trajectories. Standardized path coefficients are shown: a = association between retirement transition pattern (abrupt stop vs gradual transition) and leisure reconfiguration; b = association between leisure reconfiguration and depressive symptom trajectory; c’ = direct effect of retirement transition pattern on depressive symptom trajectory. The indirect effect (a × b = 0.13) accounted for 31.7% of the total effect. Paths from social support and psychological capital to both leisure reconfiguration and depressive symptom trajectory are also shown. ***P < .001, **P < .01. Note: Paths a and b are derived from cross-sectional, retrospective baseline data. This analysis demonstrates a statistical mediation pattern but does not establish causal mediation due to the observational design and the potential for shared antecedents.

Structural equation modeling was used to examine whether retrospectively reported reconfiguration statistically mediates the association between retirement transition patterns and adverse depressive symptom trajectories. The results are presented in Table 4, and the path diagram of the mediation analysis is shown in Figure 3. The total effect of the 2 poor transition patterns (AS and NST vs GT) on the attribution to poor trajectories was significant. For the contrast of AS versus GT, the total effect was β = 0.41 (95% CI: 0.32–0.50, *P* < .001), the direct effect was β = 0.28 (95% CI: 0.19–0.37, *P* < .001), and the total indirect effect via leisure reconfiguration was β = 0.13 (95% CI: 0.09–0.17, *P* < .001), accounting for 31.7% of the total effect. Decomposition of the overall mediation revealed that increased travel diversity post-retirement was the strongest specific mediator (indirect effect β = 0.07, 95% CI: 0.04–0.10, 17.1% mediated), followed by increased social activity participation (β = 0.04, 95% CI: 0.02–0.06, 9.8% mediated) and new skill acquisition (β = 0.02, 95% CI: 0.01–0.03, 4.9% mediated). The results of these mediation analyses are based on observational cross-sectional data on exposure associations with mediating variables and should be interpreted as suggestive of potential pathways rather than causal mediation.

**Table 4:** Statistical mediation analysis: self-reported leisure activity reconfiguration as a potential mediator between retirement transition pattern and depressive symptom trajectory (stable-low vs adverse trajectories).

Path and effect	Estimate (β)	Bootstrapped 95% CI	*P*	Proportion mediated (%)
Total effect (c)				
Abrupt stop (vs Gradual) → Adverse trajectory	0.41	(0.32–0.50)	<.001	–
Non-standard (vs Gradual) → Adverse trajectory	0.52	(0.42–0.62)	<.001	–
Direct effect (c’)				
Abrupt stop (vs Gradual) → Adverse trajectory	0.28	(0.19–0.37)	<.001	–
Non-standard (vs Gradual) → Adverse trajectory	0.33	(0.24–0.42)	<.001	–
Indirect effect (a × b) via leisure reconfiguration				
Path a: Abrupt stop → Reconfiguration score	−0.38	(−0.45 to −0.31)	<.001	–
Path b: Reconfiguration score → Adverse trajectory	−0.34	(−0.40 to −0.28)	<.001	–
Total indirect effect (a × b)	0.13	(0.09–0.17)	<.001	31.7
Specific indirect via travel diversity	0.07	(0.04–0.10)	<.001	17.1
Specific Indirect via social activity	0.04	(0.02–0.06)	<.001	9.8
Specific indirect via new skill acquisition	0.02	(0.01–0.03)	.003	4.9

Note: Adverse trajectory combines gradual-increaser, high-fluctuating, and late-onset surge classes. Standardized coefficients are reported. Bootstrap samples = 5000. The mediation model was adjusted for age, sex, education, household assets, social support, and baseline health status. Both exposure and mediator were assessed retrospectively at baseline. The mediation estimates reflect statistical mediation consistent with a potential pathway but do not establish causal mediation due to the cross-sectional assessment of the exposure-mediator relationship and possible shared antecedents.

CI = confidence interval.

### 3.5. Stratified and sensitivity analyses

**Figure 4.**
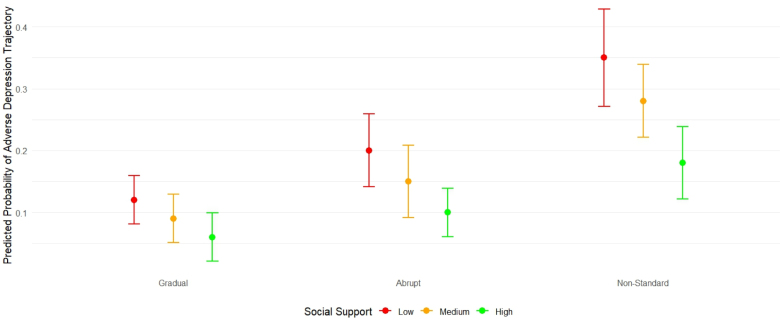
The moderating effect of social support on the association between retirement transition patterns and adverse depressive symptom trajectories. Predicted probabilities of belonging to an adverse depressive symptoms’ trajectory (any trajectory other than stable‑low) are displayed by retrospectively reported retirement transition pattern (gradual transition, abrupt stop, non‑standard transition) and social support tertile (low, medium, high). Error bars indicate 95% confidence intervals. The association between transition pattern and adverse trajectory varied significantly by social support level (interaction *P* = .008).

**Figure 5.**
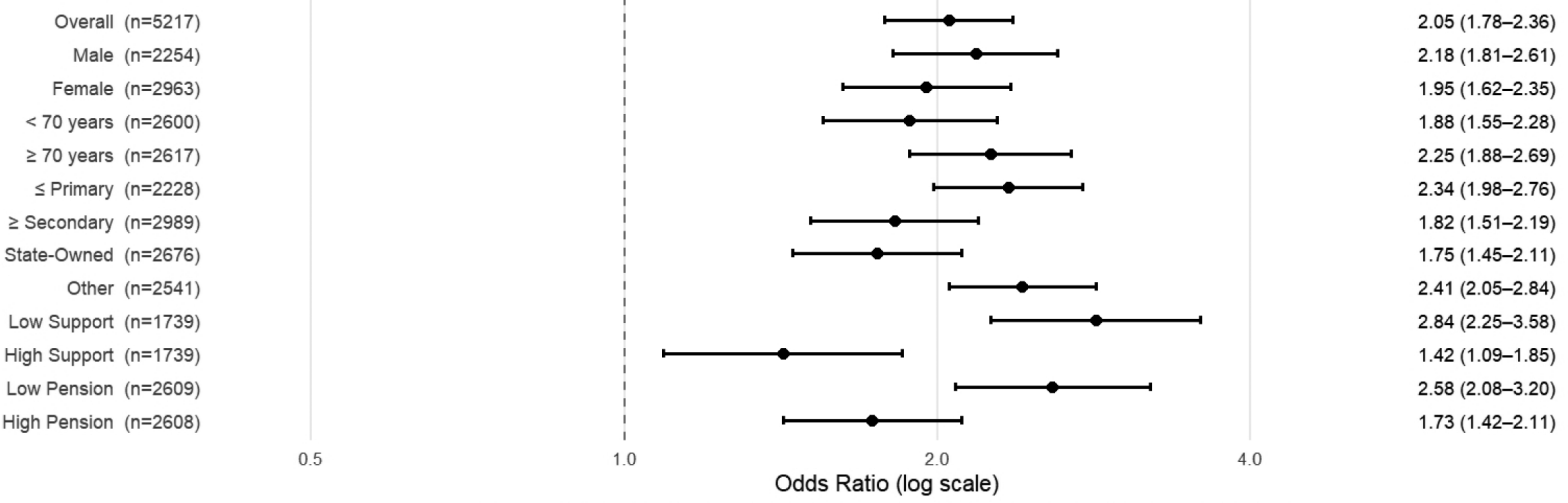
Subgroup analysis of the association between abrupt retirement transition (vs gradual transition) and adverse depressive symptom trajectory. Odds ratios (ORs) and 95% confidence intervals (CIs) were derived from multivariable logistic regression models adjusted for age, sex, education, work unit type, household assets, chronic conditions, physical activity, and baseline loneliness. The reference group is the gradual transition pattern. Sample sizes (n) for each subgroup are displayed adjacent to the subgroup labels. Higher ORs indicate greater odds of belonging to an adverse depression trajectory associated with an abrupt (vs gradual) retirement transition.

**Figure 6.**
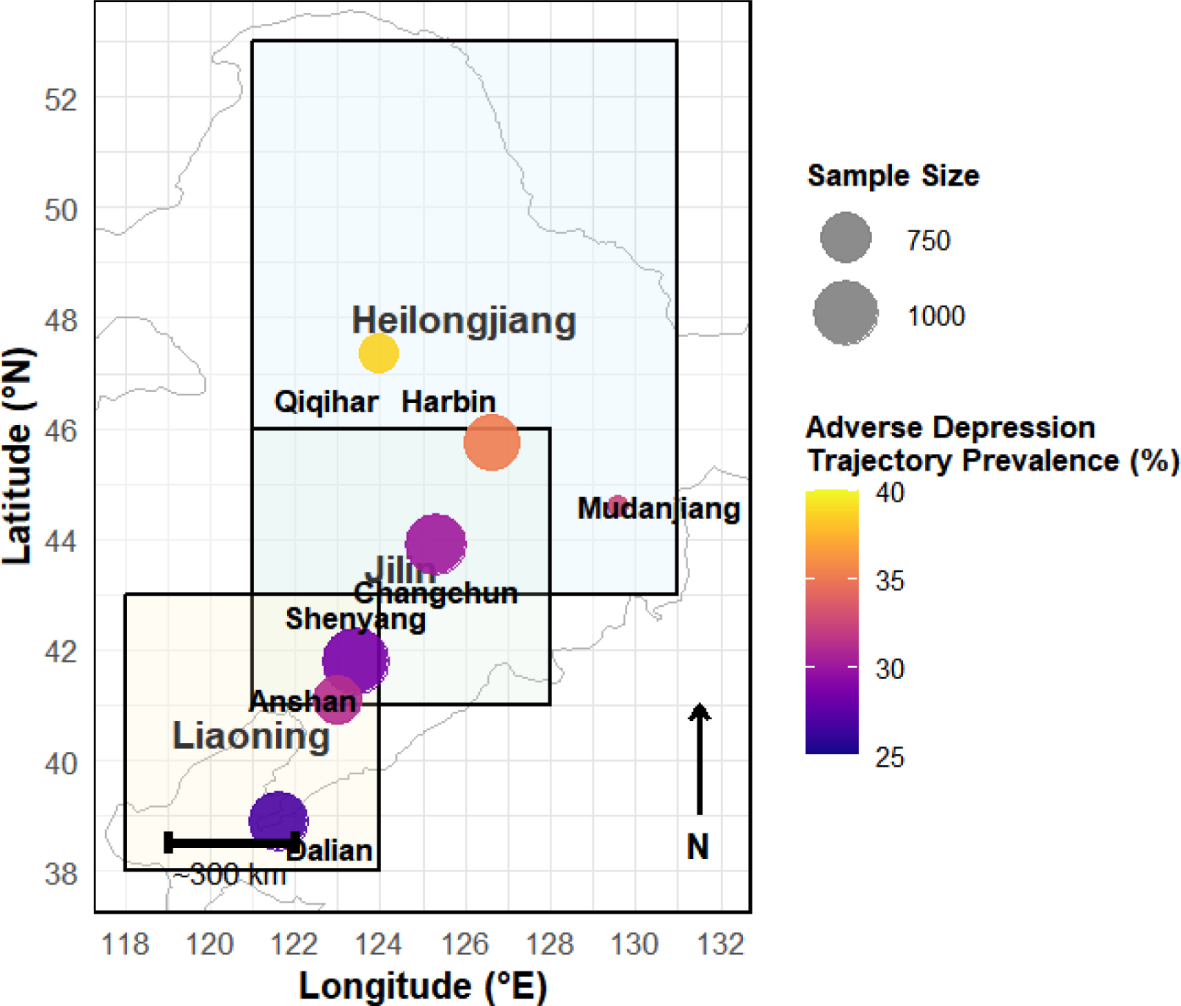
The spatial distribution of adverse depressive symptom trajectory prevalence across major cities in Northeast China. The map shows 7 major study sites in Heilongjiang, Jilin, and Liaoning provinces. Circle size represents sample size (range: 530–1050 participants per city). Circle color indicates the prevalence of adverse depressive symptom trajectories (range: 26.8–38.7%), with warmer colors indicating higher prevalence. The arrow indicates the north direction, and the scale bar represents approximately 300 km. City labels are positioned to avoid overlapping, with arrows indicating direction from data point to label when necessary.

Stratified analyses examined whether the association of retirement transition patterns with adverse depressive symptom trajectories was influenced by the level of social support and the level of economic resources (Table 5). A significant interaction was observed for both social support (*P* = .008 for interaction) and pension level (*P* = .023 for interaction). The observed association of abrupt and nonstandard retirement transitions was markedly stronger among individuals with low social support (e.g., NST aOR = 3.67, 95% CI: 2.87–4.70 in low support vs aOR = 1.68, 95% CI: 1.27–2.22 in high support) and those with low pension income (NST aOR = 3.22, 95% CI: 2.56–4.05 in low pension vs aOR = 2.04, 95% CI: 1.65–2.52 in high pension). The moderating effect of social support on the association between retirement transition patterns and adverse depressive symptom trajectories is shown in Figure 4. The adverse association of AS and nonstandard retirement was significantly stronger among individuals with lower social support (e.g., aOR = 3.67 for NST in the low support group vs 1.68 in the high support group) and was also significantly stronger among individuals with lower pension income. The results of subgroup analysis showed that the association between AS retirement and adverse depression trajectory was different in different populations, as shown in Figure 5. In Figure 5, the 2 rows of state-owned enterprises and other enterprises represent the estimated effect of state-owned enterprises or other types of individuals before retirement. The spatial distribution of adverse depressive symptom trajectories in the 3 northeastern provinces is shown in Figure 6. Sensitivity analyses, including multiple imputations for missing data and exclusion of participants depressed at baseline, yielded consistent results (sample sizes for complete-case and imputed analyses were n = 4987 and n = 5217, respectively).

**Table 5:** Stratified analysis: association between retrospectively reported retirement transition pattern and adverse depressive symptom trajectory by level of social support.

Subgroup	Gradual transition (Ref)	Abrupt stop		Non-standard transition		*P* for interaction
	aOR (95% CI)	aOR (95% CI)	*P*	aOR (95% CI)	*P*	
Overall	1.00	2.05 (1.78–2.36)	<.001	2.52 (2.16–2.94)	<.001	–
By social support tertile						
Low (score < 7)	1.00	2.84 (2.25–3.58)	<.001	3.67 (2.87–4.70)	<.001	.008
Medium (score 7–10)	1.00	1.98 (1.62–2.41)	<.001	2.38 (1.92–2.95)	<.001	
High (score > 10)	1.00	1.42 (1.09–1.85)	.009	1.68 (1.27–2.22)	<.001	
By pension level						
Low (<2000 RMB/mo)	1.00	2.58 (2.08–3.20)	<.001	3.22 (2.56–4.05)	<.001	.023
High (≥2000 RMB/mo)	1.00	1.73 (1.42–2.11)	<.001	2.04 (1.65–2.52)	<.001	
By urban/rural residence						
Urban	1.00	1.89 (1.57–2.27)	<.001	2.31 (1.89–2.83)	<.001	.134
Rural	1.00	2.38 (1.90–2.99)	<.001	2.98 (2.34–3.79)	<.001	

Note: Adverse depression trajectory is defined as membership in any trajectory other than stable-low. All models are adjusted for age, sex, education, work unit type, household asset index, number of chronic conditions, physical activity, and baseline loneliness. The interaction *P*-value tests the heterogeneity of the retirement transition effect across subgroups.

aOR = adjusted odds ratio, CI = confidence interval.

## 4. Discussion

This observational study, utilizing prospectively collected depressive symptoms data, found a significant longitudinal association between retrospectively reported retirement transition patterns and subsequent trajectories of depressive symptoms within the unique socio-historical context of post-danwei China. The central findings reveal that, compared to a report of a gradual, voluntary transition, a report of an abrupt or nonstandard retirement pattern is strongly associated with membership in more adverse trajectories of depressive symptoms in later life. This observational association was partly explained by individuals’ self-reported success in reorganizing their leisure activities after retirement and was particularly strong in groups with low social support and economic resources. These findings extend the theoretical framework by embedding life course transition theory in specific institutional legacies, providing a contextualized exploration of retirement as a process that may be associated with identity reconstruction.^[27]^

### 4.1. Theoretical integration: bridging institutional change and life course psychology

The strong association between retirement transition patterns and depressive symptoms trajectories highlights the inadequacy of viewing retirement as a unifying experience. The findings resonate with the life course perspective that emphasizes early transitions and their timing and sequencing on long-term effects.^[28]^ An AS, especially a nonstandard retirement, represents a disruptive, ill-timed transition that can drain mental resources.^[29]^ However, this study moves beyond the individual time dimension to emphasize the role of institutional pathways. The historical work-unit system provided a scripted, predictable life course. Its disintegration has created a vacuum in which transitional approaches, whether autonomously gradual or externally imposed ASs, are key determinants of late-life adaptation. This contrasts with studies in Western contexts with less rigid prior employment structures and suggests that the stability of the pre-retirement ecosystem itself is a key determinant of post-retirement resilience.^[30]^ The mediating role of leisure activity reconfiguration provides a key institutional link. This is consistent with continuity theory, but with important corrections to the theory. The theory posits that individuals preserve well-being by sustaining familiar patterns. The results suggest that in the face of a fundamental role loss, mere continuity of routines may be insufficient. Instead, active restructuring, i.e., conscious expansion and diversification of leisure activities, is essential.^[31]^ This process can be understood by the theory of selective optimal compensation. Successful aging selects new goals, optimizes resources, and compensates for lost roles due to work.^[32]^ Gradual retirement provides time and psychological space for the adaptive unfolding of this selective optimization compensation process, while an AS may overwhelm the individual’s ability to compensate, leading to maladaptive results.^[33]^

### 4.2. Unpacking the mechanisms: from institutional exit to identity reconstruction

The differential impact of retirement transition patterns on mental health trajectories may play a role through interrelated psychosocial pathways around identity, sociality, and structure. Retiring from work means not just losing a job; it means disembedding from an overarching system that once provided a coherent social identity. The abrupt termination of this role, without a transitional period of reconstruction, ruptures the individual’s self-concept and creates a vacuum of purpose and meaning, which is the core basis of depression symptomatology.^[34]^ By contrast, the GT, as an anticipatory socialization period, allows for the temporary testing and integration of the new social person identity. This process is actively facilitated by the reconfiguration of leisure activities, in which activities such as educational travel or skill-based hobbies become new vehicles for developing a sense of purpose and self-worth outside the work role.^[35]^ At the same time, the disintegration of the work-unit disintegrates a preformed, dense network of daily social interactions, which can lead to a rapid contraction into isolation. Therefore, the ability of individuals to rebuild social capital is crucial. The reconfiguration of leisure activities, especially through socially embedded activities such as group travel or community clubs, serves as a positive engine for generating new, voluntary, interest-based connections.^[36]^ This finding shows that low social support significantly exacerbates the risk of adverse depressive symptom trajectories after an AS. People with strong existing networks may be protected from social losses, while those without must urgently construct new connections, a task that becomes extraordinarily difficult without the buffer period provided by a GT.^[37]^ Another interpretive path involves the loss of externally imposed daily structures and regular experiences of competence inherent in work. The resulting anomie and self-efficacy erosion can be profound. Successful restructuring of leisure activities directly counteracts this by introducing self-constructed structures and mastering opportunities.^[38]^ Planning a complex trip or mastering a new skill provides a tangible challenge that rebuilds a sense of autonomy and regularity. The significant mediating role of travel diversity and new skill learning in our analysis underscores the importance of this mastery path. Gradual retirement provides the mental space to engage in such reconstructive projects, while an AS may trigger a state of confusion, leading to behavioral withdrawal and passivity, setting the stage for gradual or fluctuating depressive decline.^[39]^

### 4.3. Contextualizing the findings: specificity and generality

The results are both consistent with and different from international literature. The protective effect of gradual retirement is consistent with European and American studies linking phased retirement to better well-being.^[40]^ However, the strength of the association and the prominence of the nonstandard retirement group highlight a vulnerability rooted in the specific context of China’s economic transformation. The characteristics of this group reflect a generation that has endured the most market reforms and lacks the institutional shelter of the state sector. Their elevated odds of membership in the late-onset surge trajectory are particularly telling, suggesting a delayed psychological reckoning with cumulative disadvantage.^[41]^ Moreover, even after adjusting for individual socioeconomic status, there was an independent significant effect of pre-retirement work unit type, which was a unique contribution. This suggests that the type of institution an individual leaves behind imprints a cultural and resource blueprint that has implications beyond mere economic means and, in turn, retirement adaptation.^[42]^

### 4.4. Implications for a gerontological society

The implications of this research extend beyond academia to policy and practice. For policymakers, it suggests that supporting healthy aging requires a focus on the process of retirement, not just the financial outcomes. Labor and social policies that encourage and promote phased retirement options, as well as midlife planning education, can produce significant public mental health benefits by promoting better preparation.^[43]^ For community and healthcare practitioners, these findings justify the inclusion of retirement transition assessments in geriatric care to identify those who experience an AS or involuntary retirement, treat them as at elevated risk for subsequent depressive symptoms, and proactively connect them to community activities and social groups.^[44]^ It redefines the promotion of leisure activities, especially group travel and lifelong learning, not only as marginal entertainment but also as the core of preventive mental health care for new retirees.^[45]^ By understanding and supporting the journey from a work-unit member to a thriving social individual, societies can foster more resilient pathways in the increasingly lengthy later years.

### 4.5. Limitations and future directions

There are several limitations in this study. First, the assessment of the primary exposure variable (retirement transition pattern) and the key mediating variable (reconfiguration of leisure activities) relied on retrospective self-reporting at baseline. This introduces a potentially significant recall bias. For example, individuals with current depressive symptoms may be more likely to recall their retirement as an AS or involuntary and perceive their leisure as maladaptive. This may exaggerate the observed association. In addition, cross-sectional measures of exposure-mediation relationships hinder strong causal inferences about mediation pathways. The observed mediating effects may reflect common antecedents (e.g., personality traits, pre-retirement resources) rather than causal chains. Therefore, the mediation analysis should be interpreted as statistical mediation, providing evidence consistent with a potential pathway, rather than as definitive causal mediation. Second, despite adjusting for comprehensive confounders and employing sensitivity analysis (inverse probability weighting, *E*-value), there may still be residual confounding of unmeasured factors, such as personality traits (e.g., optimism, resilience, neuroticism), spousal dynamics, or detailed work history. It remains plausible that individuals who plan for gradual retirement themselves have greater psychological and social resources that independently contribute to depression prevention. Third, our measures of reconfiguration of leisure activities, although multidimensional, may fail to capture the subjective meaning and emotional participation of activities, which may be critical for mental health benefits. Fourth, the large attrition from baseline to the analyzed sample (35.4%) and the more favorable characteristics of the analyzed sample suggest a potential selection bias that may limit the generality of the findings. These limitations point out the way for future research. Prospective research should use mixed methods to track the retirement transition process in real time, combining psychological assessment and in-depth interviews to capture the subjective experience of identity change. Future work should prioritize the prospective measurement of the retirement transition and the mediating factors in its process. Intervention research is the next logical step: RCTs testing retirement transition planning workshops that specifically instruct individuals in leisure activity restructuring skills will provide causal evidence and direct practice. Importantly, as the moderation analysis indicates, such interventions should be tailored to provide intensive support to those groups with scarce social and economic resources.

## 5. Conclusion

This observational study found that retrospectively reported retirement transition patterns were significantly associated with subsequent depressive symptom trajectories in later life. Reports of abrupt or non-prescriptive retirement transitions were significantly associated with an increased likelihood of membership in adverse symptom trajectories compared with reports of gradual retirement. Self-reported successful reconfiguration of leisure activities in retirement, particularly through travel diversification and skill acquisition, was found to statistically mediate about 32% of this association. This observed association was particularly evident in groups with limited socioeconomic resources. These findings, from an observational study with retrospective exposure assessment, suggest that recalled retirement transition experiences are strongly associated with long-term patterns of mental health. The psychological impact appears to depend not only on the departure but also on the nature of the recollection of the transition and the perceived success of the reconstruction of meaning and structure thereafter. Despite limitations associated with retrospective measurement and potential confounding, these results suggest that policies and interventions designed to support GTs and promote leisure re-engagement in retirement merit further study as potential public health strategies to promote mental health in later life. Future prospective and interventional studies are needed to establish cause and effect.

## Acknowledgments

The author would like to thank all individuals who participated in this study.

## Author contributions

**Data curation:** Lanka Wu.

**Investigation:** Lanka Wu.

**Methodology:** Lanka Wu.

**Project administration:** Lanka Wu.

**Visualization:** Lanka Wu.

**Writing – original draft:** Lanka Wu.

**Writing – review & editing:** Lanka Wu.

## References

[1] Rudnicka E Napierała P Podfigurna A Męczekalski B Smolarczyk R Grymowicz M. The World Health Organization (WHO) approach to healthy ageing. Maturitas. 2020;139:6–11. 32747042 10.1016/j.maturitas.2020.05.018 PMC7250103

[2] He L Wang K Wang J. The effect of serving as a danwei leader before retirement on self-rated post-retirement health: empirical evidence from China. BMC Public Health. 2022;22:573. 35321667 10.1186/s12889-022-12937-z PMC8941785

[3] He L Wang K Li T. The positive impact of having served as a danwei leader on post-retirement life satisfaction: experiences in China. Front Psychol. 2021;12:783059. 35027903 10.3389/fpsyg.2021.783059 PMC8748259

[4] Xue B Head J McMunn A. The impact of retirement on cardiovascular disease and its risk factors: a systematic review of longitudinal studies. Gerontologist. 2020;60:e367–77. 31091304 10.1093/geront/gnz062 PMC7362617

[5] Henkens K van Dalen HP Ekerdt DJ. What we need to know about retirement: pressing issues for the coming decade. Gerontologist. 2018;58:805–12. 31287535 10.1093/geront/gnx095

[6] Hetschko C Knabe A Schöb R. Looking back in anger? Retirement and unemployment scarring. Demography. 2019;56:1105–29. 31041604 10.1007/s13524-019-00778-2

[7] Mosca I Barrett A. The impact of voluntary and involuntary retirement on mental health: evidence from older irish adults. J Ment Health Policy Econ. 2016;19:33–44. 27084792

[8] Liu H Zhou Z Fan X. Association between multiple chronic conditions and depressive symptoms among older adults in china: evidence from the China Health and Retirement Longitudinal Study (CHARLS). Int J Public Health. 2023;68:1605572. 36938299 10.3389/ijph.2023.1605572 PMC10020227

[9] Nilsen C Agahi N Shaw BA. Does the association between leisure activities and survival in old age differ by living arrangement? J Epidemiol Community Health. 2018;72:1–6. 29079586 10.1136/jech-2017-209614

[10] Zhou B Liu X Yu P. Toward successful aging: the Chinese Health Criteria for the elderly. Aging Med (Milton). 2018;1:154–7. 31942493 10.1002/agm2.12028 PMC6880742

[11] Lee S Heo J. Older women’s perspectives on leisure commitment for coping with chronic illnesses. Health Care Women Int. 2020;41:1018–35. 32870750 10.1080/07399332.2020.1799377

[12] Sjöberg O. Work-retirement transitions and mental health: a longitudinal analysis of the role of social protection generosity in 11 countries. Scand J Public Health. 2023;51:90–7. 34510984 10.1177/14034948211042130 PMC9903240

[13] Zhu Y Li C Xie W Zhong B Wu Y Blumenthal JA. Trajectories of depressive symptoms and subsequent cognitive decline in older adults: a pooled analysis of two longitudinal cohorts. Age Ageing. 2022;51:afab191. 34657957 10.1093/ageing/afab191

[14] Zhang X Yang Y He L Wang Z. Fading authority, rising depression: occupational identity and mental health among China’s retired danwei leaders. Front Psychiatry. 2025;16:1663695. 41367944 10.3389/fpsyt.2025.1663695 PMC12682773

[15] Huang T Lyu H Chen X Ren J. The relationship between sense of community and general well-being of Chinese older adults: a moderated mediation model. Front Psychol. 2022;13:1082399. 36687867 10.3389/fpsyg.2022.1082399 PMC9859671

[16] Wagner C Carmeli C Jackisch J. Life course epidemiology and public health. Lancet Public Health. 2024;9:e261–9. 38553145 10.1016/S2468-2667(24)00018-5

[17] Glasner T van der Vaart W. Applications of calendar instruments in social surveys: a review. Qual Quant. 2009;43:333–49. 20046840 10.1007/s11135-007-9129-8 PMC2798968

[18] Dingemans E Henkens K. How do retirement dynamics influence mental well-being in later life? A 10-year panel study. Scand J Work Environ Health. 2015;41:16–23. 25311938 10.5271/sjweh.3464

[19] Hess M Naegele L Mäcken J. Attitudes towards working in retirement: a latent class analysis of older workers’ motives. Eur J Ageing. 2021;18:357–68. 34483800 10.1007/s10433-020-00584-5 PMC8377099

[20] Akbayrak E Powell PA Tunc N Barnes S. The relationship between subjective cognitive decline and cognitive leisure activity engagement: a systematic review. Gerontologist. 2024;65:gnae176. 39657691 10.1093/geront/gnae176 PMC11772863

[21] Zhang Y Hoozemans M Pijnappels M Bruijn SM. A formula for calculating 30-item Geriatric Depression Scale (GDS-30) scores from the 15-item version (GDS-15). Exp Gerontol. 2023;172:112077. 36587798 10.1016/j.exger.2022.112077

[22] Barrette E Higuera L Wherry K. Assessment of the E-value in the presence of bias amplification: a simulation study. BMC Med Res Methodol. 2024;24:79. 38539082 10.1186/s12874-024-02196-4 PMC10976722

[23] Tennant PWG Murray EJ Arnold KF. Use of directed acyclic graphs (DAGs) to identify confounders in applied health research: review and recommendations. Int J Epidemiol. 2021;50:620–32. 33330936 10.1093/ije/dyaa213 PMC8128477

[24] Igartua JJ Hayes AF. Mediation, moderation, and conditional process analysis: concepts, computations, and some common confusions. Span J Psychol. 2021;24:e49. 35923144 10.1017/SJP.2021.46

[25] Austin PC Stuart EA. Moving towards best practice when using inverse probability of treatment weighting (IPTW) using the propensity score to estimate causal treatment effects in observational studies. Stat Med. 2015;34:3661–79. 26238958 10.1002/sim.6607 PMC4626409

[26] Chung WT Chung KC. The use of the E-value for sensitivity analysis. J Clin Epidemiol. 2023;163:92–4. 37783401 10.1016/j.jclinepi.2023.09.014

[27] Wood RE Pachana NA. The role of meaning in the retirement transition: scoping review. Gerontologist. 2025;65:gnaf076. 39969022 10.1093/geront/gnaf076 PMC12082295

[28] Elder GH Shanahan MJ Jennings JA. Human Development in Time and Place. John Wiley & Sons, Inc. 2015.

[29] Chan MCH Chung EKH Yeung DY. Attitudes toward retirement drive the effects of retirement preparation on psychological and physical well-being of hong kong chinese retirees over time. Int J Aging Hum Dev. 2021;93:584–600. 32468835 10.1177/0091415020926843

[30] Scherger S. Flexibilizing the retirement transition: why, how and for whom? Conceptual clarifications, institutional arrangements and potential consequences. Front Sociol. 2021;6:734985. 34708108 10.3389/fsoc.2021.734985 PMC8542977

[31] Tiilikainen E Hujala A Kannasoja S Rissanen S Närhi K. “They’re always in a hurry” - Older people´s perceptions of access and recognition in health and social care services. Health Soc Care Community. 2019;27:1011–8. 30723951 10.1111/hsc.12718

[32] Freund AM Baltes PB. The Orchestration of Selection, Optimization, and Compensation: An Action-Theoretical Conceptualization of a Theory of Developmental Regulation. In: Control of Human Behavior, Mental Processes, and Consciousness. 2012:35 –58.

[33] Bačová V Halama P Kordačová J. Irrational beliefs indirectly predict retirement satisfaction through the conceptualization of retirement: a cross-sectional study in a sample of recent retirees. BMC Psychol. 2023;11:195. 37403131 10.1186/s40359-023-01237-9 PMC10318810

[34] Haslam C Lam BCP Ghafoori E. A longitudinal examination of the role of social identity in supporting health and well-being in retirement. Psychol Aging. 2023;38:615–26. 37307317 10.1037/pag0000757

[35] Nimrod G Shrira A. The paradox of leisure in later life. J Gerontol B Psychol Sci Soc Sci. 2016;71:106–11. 25315158 10.1093/geronb/gbu143 PMC4861252

[36] Huxhold O Fiori KL Webster NJ Antonucci TC. The strength of weaker ties: an underexplored resource for maintaining emotional well-being in later life. J Gerontol B Psychol Sci Soc Sci. 2020;75:1433–42. 32055856 10.1093/geronb/gbaa019 PMC7424273

[37] Litwin H Levinsky M. Social networks and mental health change in older adults after the Covid-19 outbreak. Aging Ment Health. 2022;26:925–31. 33749448 10.1080/13607863.2021.1902468

[38] Stephan Y Sutin AR Luchetti M Terracciano A. Feeling older and the development of cognitive impairment and dementia. J Gerontol B Psychol Sci Soc Sci. 2017;72:966–73. 27436103 10.1093/geronb/gbw085 PMC5927095

[39] Balogh R Gadeyne S Vanroelen C Warhurst C. Multidimensional employment trajectories and dynamic links with mental health: evidence from the UK Household Longitudinal Study. Scand J Work Environ Health. 2025;51:26–37. 39476405 10.5271/sjweh.4193 PMC11697615

[40] Vanajan A Bültmann U Henkens K. Do older manual workers benefit in vitality after retirement? Findings from a 3-year follow-up panel study. Eur J Ageing. 2021;18:369–79. 34483801 10.1007/s10433-020-00590-7 PMC8377110

[41] Kendig H Gong CH Yiengprugsawan V Silverstein M Nazroo J. Life course influences on later life health in China: childhood health exposure and socioeconomic mediators during adulthood. SSM Popul Health. 2017;3:795–802. 29349264 10.1016/j.ssmph.2017.10.001 PMC5769110

[42] Zhang A Zhang Y Tao Y. Does retirement make people happier? Evidence from China. Front Public Health. 2022;10:874500. 35784195 10.3389/fpubh.2022.874500 PMC9247314

[43] Wang T Liu H Zhou X Wang C. The effect of retirement on physical and mental health in China: a nonparametric fuzzy regression discontinuity study. BMC Public Health. 2024;24:1184. 38678184 10.1186/s12889-024-18649-w PMC11520119

[44] Rafalski JC Noone JH O’Loughlin K de Andrade AL. Assessing the process of retirement: a cross-cultural review of available measures. J Cross Cult Gerontol. 2017;32:255–79. 28516309 10.1007/s10823-017-9316-6

[45] Li H Zeng W. Is leisure sedentary time associated with mental health issues? Evidence from China Health and Nutrition Survey. Front Public Health. 2025;13:1517830. 39980927 10.3389/fpubh.2025.1517830 PMC11839644

